# Color stability of resin composites for orthodontic attachments: an in vitro study

**DOI:** 10.1590/2177-6709.27.1.e2220432.oar

**Published:** 2022-04-11

**Authors:** Vitória de Oliveira CHAMI, Fabiane GEBERT, Débora do Canto ASSAF, Anna Carolina Teixeira CENTENO, Vilmar Antônio FERRAZZO, Leticia Brandão DURAND, Mariana MARQUEZAN

**Affiliations:** 1 Department of Orthodontics, School of Dentistry, Graduate Program, Federal University of Santa Maria, (Santa Maria/RS, Brazil); 2 Clinical practice (Primavera do Leste/MT, Brazil); 3 Department of Stomatology, School of Dentistry, Federal University of Santa Maria (Santa Maria/RS, Brazil); 4 Department of Operative Dentistry, School of Dentistry, Federal University of Santa Maria (Santa Maria/RS, Brazil)

**Keywords:** Color, Composite resins, Orthodontic appliances, Removable

## Abstract

**Objective:**

The aim of the present study was to evaluate the color stability of Filtek Z350 XT, Filtek Z250 XT, Z100 resin composites and Transbond XT orthodontic resin, all used in orthodontic attachments, when immersed in popular beverages.

**Methods:**

Thirty disk-shaped specimens of each resin composite (2 x 5mm) were manufactured and randomly divided into six groups according to immersion solutions: coffee, red wine, white wine, regular beer, dark beer and deionized water (control). The specimens were fully immersed in each of the solutions for six days at 37°C, representing approximately six months of consumption. The color measurements were evaluated by a reflection spectrophotometer, at baseline (before immersion) and after staining. L*a*b* coordinates were measured and the color change (ΔE_00_) was calculated using the CIEDE2000 formula. The data were analyzed by ANOVA/Tukey tests at a significance level of 0.05.

**Results:**

The resin composites immersed in white wine and regular beer showed either imperceptible or clinically acceptable ΔE_00_, and no difference from the control group (*p*= 0.4449 and *p*= 0.467 respectively). Immersion in coffee and red wine were considered clinically unacceptable and were significantly different from the control group (*p*= 0.0028 and *p*= 0.0475 respectively).

**Conclusion:**

Based on the results of the present study, the consumption of coffee and red wine may cause color change of the resin composite attachments above the visual acceptability threshold, and impair aesthetics during treatment.

## RESUMO

**Objetivo:**

O objetivo do presente estudo foi avaliar a estabilidade de cor das resinas compostas Filtek Z350 XT, Filtek Z250 XT, Z100 e resina ortodôntica Transbond XT, utilizadas para *attachments* ortodônticos, quando imersas em bebidas populares.

**Métodos:**

Trinta corpos de prova em forma de disco de cada resina composta (2 x 5mm) foram confeccionados e divididos aleatoriamente em seis grupos, de acordo com as soluções de imersão: café, vinho tinto, vinho branco, cerveja clara, cerveja escura e água deionizada (controle). Os discos foram totalmente imersos nas soluções por seis dias a 37°C, representando aproximadamente seis meses de consumo. As medidas de cor foram realizadas por um espectrofotômetro de reflexão, no *baseline* (antes da imersão) e após o manchamento. As coordenadas L*a*b* foram medidas e a variação de cor (ΔE_00_) foi calculada usando a fórmula CIEDE2000. Os dados foram analisados utilizando a análise de variância seguida pelo teste *post-hoc* de Tukey, com nível de significância de 0,05.

**Resultados:**

As resinas imersas em vinho branco e cerveja clara apresentaram ΔE_00_ imperceptível ou clinicamente aceitável, sem diferença em relação ao grupo controle (*p*= 0,44449 e *p*= 0,467, respectivamente). A imersão em café e vinho tinto foi considerada clinicamente inaceitável e significativamente diferente do grupo controle (*p*= 0,0028 e *p*= 0,0475, respectivamente).

**Conclusão:**

Com base nos resultados do presente estudo, o consumo de café e vinho tinto pode causar alteração na cor dos *attachments* ortodônticos acima do limite de aceitabilidade visual e prejudicar a estética durante o tratamento.

## INTRODUCTION

The quest for improved aesthetics during orthodontic treatment has increased the number of treatments performed with clear aligners. This technology emerged the late 20^th^ century as an alternative to conventional orthodontic treatment, and has been widely used worldwide, especially in the last ten years. In addition to aesthetics, their main advantages are removal during eating and cleaning of teeth, this way, clear aligners are better for periodontal health than fixed appliances.^1^Furthermore, patients treated with clear aligners appear to feel lower levels of pain during the first days of treatment.^2^

Attachments are one of the components often required to increase the predictability and efficiency of tooth movement during treatment with clear aligners.^3^ These are additions of resin composite bonded to the tooth enamel, to create an anchor point for the aligner. Their design is predefined during the planning phase, and must not be modified after bonding. In this case, the resin composite should not undergo the traditional polishing process.^4^

Resin composites are porous materials that may absorb coloring agents from food.^5^ Both *in vivo* and *in vitro* studies have been carried out to evaluate the color stability of resin composites used for restorations. Surface roughness can contribute to greater staining of resin composites, and rougher surfaces have a positive correlation with the color change of resin composites.^6^

Patients who seek treatment with clear aligners have greater aesthetic demands;^7^ thus, the staining of attachments could lead to dissatisfaction during orthodontic correction. Orthodontic attachments should not be polished, and consequently, may be more susceptible to staining. Therefore, the objective of this study was to determine the color stability of Filtek Z350 XT (color: A1E), Filtek Z250 XT (color: A1), and Z100 (color: A1) resin composites, and Transbond XT orthodontic resin (single color), used for orthodontic attachments, when fully immersed in coffee, red wine, white wine, regular beer, dark beer, compared to deionized water (control). These beverages were selected because they are commonly found in patients’ diets. The null hypothesis was that the tested resin composites would not present visually unacceptable staining after immersion in different beverages.

## MATERIAL AND METHODS

A total of 120 disk-shaped specimens 5-mm in diameter and 2-mm thick were made using three restorative resin composites and an orthodontic resin composite, as shown in Table 1. The specimens were manufactured with a metallic matrix in which the resin was accommodated, gently pressed between two glass plates (Jon, São Paulo, SP, Brazil) and interposed between two polyester strips (Fava, Pirituba, SP, Brazil). The resin specimens were light-cured for 40 seconds (20 seconds on each side) using an LED device (Radii-cal - SDI, Victoria, Australia), with light intensity of > 1000 mW/cm², measured by the internal radiometer of the device.

**Table 1:** Material specifications, manufacturers’ information, color, particle size, composition and batch number.

Resin composite	Manufacturer	Color	Particle size/Composition	Batch number
Filtek Z350 XT	3M ESPE, St. Paul, MN, USA	A1E	Zirconia: 4-11nm, Silica: 20nm, Bis-GMA, UDMA, TEGDMA, PEGDMA, Bis-EMA.	958068
Filtek Z250 XT	3M ESPE, St. Paul, MN, USA	A1	Zirconia/Silica: 3µm, Silica: 20nm, Bis-GMA, UDMA, Bis-EMA, PEGDMA, TEGDMA.	813334
Z100	3M ESPE, St. Paul, MN, USA	A1	Zirconia/Silica: 0,6µm, Bis-GMA, TEGDMA.	1812900649
Transbond XT	3M ESPE, St. Paul, MN, USA	Single	Silanized Silica, Bis-GMA, TEGDMA.	N912880

Abbreviations: Bis-GMA = bisphenol-A-glycidyl methacrylate, UDMA = urethane dimethacrylate,TEGDMA = triethylene glycol dimethacrylate, PEGDMA = polyethylene glycol dimethacrylate, Bis-EMA = ethoxylated bisphenol-A-dimethacrylate.

Thirty specimens of each of the four resins were divided into six subgroups to receive the following immersion solutions (n = 5): coffee, red wine, white wine, regular beer, dark beer and deionized water (control), as described in Table 2. The color measurements of all the resin composite disks were measured on a white background by a reflection spectrophotometer equipment (SP60, EX-Rite, Grand Rapids, MI, USA), according to the CIEL*a*b* parameters, at baseline (before immersion) and after staining, in an environment illuminated with natural light.

**Table 2:** Staining solutions and manufacturers’ information.

Immersion solution	Manufacturer
Red wine	Salton, Bento Gonçalves, RS, BR
Coffee	Melitta, São Paulo, SP, BR
Dark beer	Brahma, Rio de Janeiro, RJ, BR
Regular beer	Brahma, Rio de Janeiro, RJ, BR
White wine	Salton, Bento Gonçalves, RS, BR
Deionized water (control)	SS Plus, Maringá, PR, BR

The specimens were placed in lid-fitted plastic boxes, fully immersed in the solutions corresponding to each group, in an incubator at 37°C, and remained there for a period of six days. The immersion solutions were changed daily to avoid contamination by bacteria or yeast. According to Ertas et al,^8^ a day of immersion in an incubator is equivalent to a one month of consumption. Thus, six days of immersion simulated six months of consumption of each beverage. After six days, the specimens were washed with abundant water and dried with absorbent paper, to avoid dehydration, then the final color measurement of the resin composites was performed, using the same spectrophotometer and parameters.

The CIEDE2000 color-difference formula is based on the CIE L^*^a^*^b^*^ color space.^9^ ^,^ ^10^ The color change (ΔE_00_) between baseline and after six days of immersion in the beverages was calculated using the CIEDE2000 formula:

$$\Delta E_{00}=\;{\left[{\left(\Delta L’/\;K_{L}S_{L}\right)}^{2}+\;{\left(\Delta C’/\;K_{C}S_{C}\right)}^{2}\;+\;{\left(\Delta H’/\;K_{H}S_{H}\right)}^{2}+\;R_{T}\left(\Delta C’/\;K_{C}S_{C}\right)\;\left(\Delta H’/\;K_{H}S_{H}\right)\right]}^{1/2}$$

where: ΔL’, ΔC’ and ΔH’ refer to lightness, chroma, and hue differences between color measurements, respectively. K_L_, K_C_ and K_H_ are the parametric factors for the conditions and illumination influence. R_T_ (rotation function) is responsible for the interaction of hue and chroma differences in the blue region. S_L_, S_C_ and S_H_ are the weighting functions for the color difference adjustment, considering the location variation of the L*a*b* coordinates.^9^ ^,^ ^10^

The visual acceptability and perceptibility thresholds described by Paravina et al^11^ were used to interpret the results. A value of ΔE_00_ ≤ 0.8 is considered clinically imperceptible; 0.8 < ΔE_00_ ≤ 1.8 is perceptible, but clinically acceptable; ΔE_00_ > 1.8 is unacceptable; considering 1.8 < ΔE _00_ ≤ 3.6 is moderately unacceptable; 3.6 < ΔE ≤ 5.4 is clearly unacceptable, and ΔE > 5.4 is extremely unacceptable. The data were subjected to analysis of variance (ANOVA) and the Tukey *post-hoc* test, and the significance level was set at 0.05. 

## RESULTS

The color change averages (ΔE_00_) and the standard deviations of all the specimens are shown in Table 3. The ​​ΔE_00_ values ranged from 0.52 for deionized water (control) in the Z100 resin group, considered clinically imperceptible, to 16.74 for red wine in the Filtek Z350 XT resin group, considered extremely unacceptable. The color change of each of the six beverages tested with the exact same resin composite are shown in uppercase letters in the same column, with a 95% statistical difference. The color change of each beverage for the four different resins is in lowercase letters in the same line. 

**Table 3:** Means and standard deviations of ΔE_00_* values for the resin composites in the different immersion solutions.

Resin composite / Immersion solution	Filtek Z350 XT	Filtek Z250 XT	Z100	Transbond XT	*p* **-value**
Red wine	16.74 (5.85) ^A, a^	12.37 (1.73) ^A, a^	12.27 (1.03) ^A, a^	5.54 (1.91) ^A, c^	0.0475*
Coffee	10.78 (2.99) ^B, a^	10.47 (2.40) ^A, ab^	7.27 (1.83) ^B, bc^	5.40 (0.72) ^A, c^	0.0028*
Dark beer	2.94 (0.85) ^C, a^	1.38 (1.10) ^B, b^	2.77 (0.76) ^C, a^	0.90 (0.38) ^B, b^	0.0022*
Regular beer	1.56 (0.68) ^C, a^	1.62 (0.54) ^B, a^	1.92 (0.82) ^CD, a^	1.16 (0.75) ^B, a^	0.4670
White wine	1.17 (0.59) ^C, a^	1.66 (1.33) ^B, a^	0.69 (0.63) ^D, a^	1.53 (1.20) ^B, a^	0.4449
Deionized water (control)	1.20 (0.74) ^C, a^	1.72 (0.57) ^B, ab^	0.52 (0.34) ^D, b^	1.14 (0.78) ^B, ab^	0.5000
*p*-value	< 0.0001*	< 0.0001*	< 0.001*	< 0.0001*	-

*Statistical significance according to ANOVA and Tukey, p<0.05. Different uppercase letters in the same column represent statistical difference with 95% confidence. Different lowercase letters in the same line represent statistical difference with 95% confidence.

Transbond XT resin group presented the lowest ΔE_00_ among the resin composites when immersed in red wine and coffee, and was statistically significant (*p*= 0.0475, *p*= 0.0028, respectively). Filtek Z250 XT and Transbond XT resin groups (*p*= 0.0022) presented the lowest ΔE_00_ when immersed in black beer. No statistical differences were found among the four different resins among the other beverages. 

**Figure 1:**
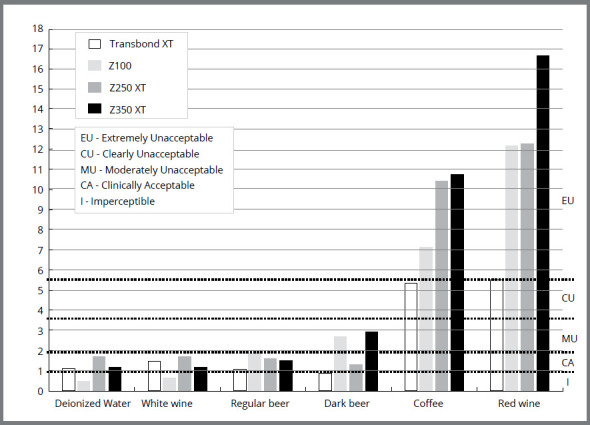
Cutoff points and ratings for visual acceptability and perceptibility thresholds for ΔE_00_** of composite resins in different immersion solutions.

Figure 1 illustrates the cutoff points and ratings for visual acceptability and perceptibility thresholds^11^ for each resin composites in different immersion solutions. Deionized water (control), white wine and regular beer showed an imperceptible or clinically acceptable standard of acceptability, and were considered clinically acceptable in the tested resin composites, except for Z100 in regular beer, which showed a slightly to moderately unacceptable color difference. The resins yielded an extremely unacceptable pattern for coffee and red wine, except Transbond XT resin in coffee, which was considered moderately unacceptable. Black beer showed a clinically acceptable standard for Transbond XT and Filtek Z250 XT resins, and moderately unacceptable standard for Z100 and Filtek Z350 XT.

The sample size requirements were evaluated according to the power calculation for this study’s sample. The calculation took into account an alpha error probability of 0.05 and mean color changes among all resin groups before and after immersion in test solutions, resulting in a sample power that ranged from 97% to 100%.

## DISCUSSION

This study was undertaken to evaluate the color stability of different resin composites used for orthodontic attachments in clear aligners treatment, simulating a clinical situation of approximately six months of consumption of different popular beverages. The null hypothesis was rejected because all of the resin composites presented visual unacceptable color change when immersed in red wine and coffee.

The visual interpretation of the staining was made by the perceptibility and acceptability thresholds for ΔE_00_. It is currently accepted that color changes of ΔE_00_ ≤ 0.8 are considered clinically imperceptible, and 0.8 < ΔE_00_≤ 1.8 are perceptible, but clinically acceptable. However, values of ΔE_00_> 1.8 are clinically unacceptable, and are divided into three types: type (a), 1.8 < ΔE_00_≤ 3.6, moderately unacceptable; type (b), 3.6 < ΔE_00_≤ 5.4, clearly unacceptable; and type (c), ΔE_00_ > 5.4, extremely unacceptable.^11^

Resin composites immersed in coffee showed extremely unacceptable values in the present study, with ΔE_00_ significantly higher than deionized water (control) (*p*= 0.0028), ranging between 10.78 and 5.40, except Transbond XT resin group, which was considered moderately unacceptable. Choi et al.^12^ and Ardu et al.^5^ tested resin composites immersed in coffee and other beverages. Color change (ΔE_00_) was considered extremely unacceptable in both studies in nine different resin composites. 

In the present study, red wine promoted highest color change in resin composites, corroborating the studies by Mundim et al.^13^, Llena et al.^14^ and Schoroeder et al.^15^ ΔE_00_ of red wine was significantly higher than deionized water (control) (*p*= 0.0475), ranging from 5.54 to 16.74. Moreover, acidic pH, water-soluble coloring agents, and alcohol have been related to resin composite staining.^5^ ^,^ ^16^

Although the specimens did not present coloring particles, this color change may be related to sorption and hydrolysis, which are characteristics of the monomers used in the resin composites.^17^ The pigmentation ability of the resin composite is directly connected to its hydrophilic properties. The more hydrophilic the resin, the greater its ability to absorb not only water, but also water-soluble coloring agents^18^. Water may conduct the staining agent towards the material, resulting in more intense staining of resin composites.^19^

Clear aligner technology is increasingly specialized and the attachments should be added to the tooth surface not only to enhance aligners retention, but also to promote difficult tooth movements.^20^ The forces and movements required for malocclusion correction are generated by the difference between the shape of attachment, clear aligner and teeth.^21^

The ability of resin composites used in attachments to prevent shape and surface alteration during six months of treatment was evaluated by Barreda et al.^4^, demonstrating that different resin composites could affect the surface, but not the shape of the attachments. In the presen study, Filtek Z350 XT resin composite revealed less surface wear, in agreement with Feinberg et al.^22^ Other study demonstrated that the use of resin composites with different viscosities does not influence the shape and volume of attachments.^23^

According to a recent systematic review, treatment with clear aligners is still deficient in controlling anterior extrusion, anterior buccolingual inclination, and is not effective in controlling rotation of rounded teeth, in particular.^24^ However, the attachments are continually being modified to increase the range of tooth movements that can be achieved,^25^ therefore, the importance of studying the resin composites used for this purpose.

As a strength of this study, the evaluation of the color change was conducted with CIEDE2000, since it has shown better adjustment potential, compared with the previous method, CIE L*a*b*. These two methods showed a statistically significant difference in measuring the perceptibility and the acceptability of the color of restorative materials.^9^ ^,^ ^10^ Another strength was the use of unpolished samples to simulate the conditions of clinical use of the resin composites in attachments, and especially considering a study by Duc et al,^6^ in which unpolished resins composites showed 30% greater color change. This greater variation can be explained by the presence of free radicals on the unpolished surface.^6^

As a limitation of this study, it should be pointed that it’s an *in vitro* study and only the color stability of resin composites was taken into account, thus the extrapolation to clinical practice should be considered with caution. The six days immersion, for example, was based on the study of Ertas et al,^8^ who estimated that 24 hours immersion was equivalent to a one month of consumption. This calculation was based on the assumption that the overall coffee intake of the population is approximately 3.2 cups/day with 15 min/cup drinking time. However, this assumption is related exclusively with coffee consumption, without considering the overall frequency of consumption of the other immersion solutions. Moreover, the surface interactions between saliva and other beverages, that may slow down or diminish the staining process, was not considered. 

Additionally, the results of the present study addressed the evaluation of Transbond XT, an orthodontic resin, available in the majority of orthodontic offices, which presented reduced staining when compared to the other resin composites. Furthermore, color stability and other factors, such as physical properties, must be taken into account when choosing the resin to be used. Thus, it is suggested that further studies should be conducted to assess the physical properties of resin composites used in attachments, such as surface wear and resistance to bonding throughout the orthodontic treatment.

## CONCLUSION

Based on the results of the present study, it may be concluded that the consumption of coffee and red wine may cause staining of the resin composite attachments above the visual acceptability threshold and impair aesthetics during treatment.
